# Prehospital lactate improves prediction of the need for immediate interventions for hemorrhage after trauma

**DOI:** 10.1038/s41598-019-50253-6

**Published:** 2019-09-24

**Authors:** Hiroshi Fukuma, Taka-aki Nakada, Tadanaga Shimada, Takashi Shimazui, Tuerxun Aizimu, Shota Nakao, Hiroaki Watanabe, Yasuaki Mizushima, Tetsuya Matsuoka

**Affiliations:** 1 Senshu Trauma and Critical Care Center, 2-23 Rinku Orai Kita, Osaka, 598-8577 Japan; 2 Chiba University Graduate School of Medicine, Department of Emergency and Critical Care Medicine, 1-8-1 Inohana, Chuo, Chiba 260-8677 Japan; 3 Center for Frontier Medical Engineering, Chiba University, 1-8-1 Inohana, Chuo Chiba, 260-8677 Japan; 4 Shimane University Faculty of Medicine, Department of Acute Care Surgery, 89-1 Enya-cho, Izumo, Shimane 693-8501 Japan

**Keywords:** Outcomes research, Preclinical research

## Abstract

The blood lactate level is used to guide the management of trauma patients with circulatory disturbance. We hypothesized that blood lactate levels at the scene (Lac *scene*) could improve the prediction for immediate interventions for hemorrhage. We prospectively measured blood lactate levels and assessed retrospectively in 435 trauma patients both at the scene and on arrival at the emergency room (ER) of a level I trauma center. Primary outcome was immediate intervention for hemorrhage defined as surgical/radiological intervention and/or blood transfusion within 24 h. Physiological variables plus Lac *scene* significantly increased the predictive value for immediate intervention (area under the curve [AUC] 0.882, 95% confidence interval [CI] 0.839–0.925) compared to that using physiological variables only (AUC 0.837, 95% CI 0.787–0.887, *P* = 0.0073), replicated in the validation cohort (n = 85). There was no significant improvement in predicting value of physiological variables plus Lac *scene* for massive transfusion compared to physiological variables (AUC 0.903 vs 0.895, *P* = 0.32). The increased blood lactate level per minute from scene to ER was associated with increased probability for immediate intervention (*P* < 0.0001). Both adding Lac *scene* to physiological variables and the temporal elevation of blood lactate levels from scene to ER could improve the prediction of the immediate intervention.

## Introduction

Immediate surgical/radiological intervention for hemostasis is a key component of management in hemorrhagic trauma^1,2^. Immediate blood transfusion to maintain hemodynamics and adequate tissue oxygenation is often needed^3^. If the need for hemostatic intervention and transfusion is accurately predictable at the scene from which emergency medical services (EMS) transports a patient to a trauma center, more preparation time to ensure earlier initiation of interventions would be available. Thus, development of a precise predictive algorithm for the need for immediate intervention for hemostasis may contribute to improvement of trauma care and better outcomes.

Predictive algorithms for blood transfusion have been studied in trauma^4–6^. In particular, prediction of the need for massive transfusion, i.e., ≥10 units of packed red blood cells (RBCs) within 24 h^7^, has been extensively studied^4,5^. Precise predictive algorithms for the need for massive transfusion using physiological variables without blood tests have been well established^4,5,8^. In contrast, less attention has been devoted to development of precise predictive algorithms for the need for immediate hemostatic interventions and transfusion.

The blood lactate level reflects poor tissue perfusion and is used to guide the management of with patients with circulatory disturbance in trauma^9^. Portable devices enable point-of-care lactate testing even in prehospital settings^10,11^. The potential utility of measuring blood lactate levels in prehospital settings was reported in patients with circulatory, respiratory, or neurological disorders who required urgent ambulance dispatch^12^.

Thus, we hypothesized that blood lactate levels at the scene could improve the prediction of need for immediate interventions for hemorrhage after hospital arrival. We prospectively measured blood lactate levels in trauma patients both at the scene and on arrival at a level I regional trauma center. Primary outcome was immediate intervention for hemorrhage defined as immediate surgical/radiological intervention for hemostasis and/or blood transfusion within 24 h after emergency room (ER) arrival. The amount of change per minute in blood lactate levels taken at the scene and on ER arrival was further evaluated.

## Results

### Baseline patient characteristics and outcomes

In derivation cohort 1, patients in the case group were significantly older, with higher probability of penetrating injury, higher injury severity score, higher blood lactate levels on scene (Lac *scene*) and in the ER (Lac *emergency room*), and worse physiological data compared to the control group (Table 1). Patients in the case group had increased 28-day mortality and longer intensive care unit stay compared to the control group (*P* < 0.0001).

**Table 1:** Baseline patient characteristics and clinical outcomes in cohort 1.

Characteristics or outcomes	Case^α^ (n = 78)	Control (n = 272)	*P*-values
Age, years	62 (41–75)	39 (21–55)	<0.0001
Male, n (%)	51 (65.4)	189 (69.5)	0.58
**Mechanism of injury**			
Penetrating, n (%)	5 (6.4)	5 (1.8)	0.048
Blunt, n (%)	73 (93.6)	267 (98.2)	0.30
Road injury, n (%)	57 (73.1)	224 (82.4)	
Fall, n (%)	10 (12.8)	26 (9.6)	
Compression machinery, n (%)	4 (5.1)	6 (2.2)	
Other, n (%)	2 (2.6)	11 (4.0)	
**Physiological data** ^**β**^			
Systolic blood pressure, mm Hg	126 (101–145)	131 (118–146)	0.069
Heart rate, beats/min	92 (80–114)	85 (74–97)	0.0014
Respiratory rate, breaths/min	25 (20–30)	21 (16–25)	0.0010
Glasgow Coma Scale	13 (8–14)	14 (13–15)	<0.0001
Shock index	0.75 (0.57–1.01)	0.64 (0.54–0.77)	0.0003
**Lactate**			
Scene, mg/dL	3.1 (2.3–4.7)	2.0 (1.6–2.6)	<0.0001
Emergency room, mg/dL	3.0 (2.3–5.0)	1.8 (1.3–2.5)	<0.0001
Delta^†^, delta/min	0.010 (−0.027–0.059)	−0.0071 (−0.028–0.022)	0.030
Scene to hospital, min	22 (14–29)	18 (13–25)	0.069
Injury severity score	27 (19–40)	9 (1–17)	<0.0001
Positive FAST exam^‡^, n (%)	10 (12.8)	5 (1.8)	0.0002
Blood transfusion, n (%)	65 (83.3)	—	—
Red blood cells^§^, mL	560 (0–1120)	—	—
Fresh frozen plasma^¶^, mL	1040 (480–2060)	—	—
Massive transfusion, n (%)	28 (35.9)	—	
**Hemostatic intervention**			
Surgery, n (%)	12 (15.4)	—	—
IVR, n (%)	28 (35.9)	—	—
Both, n (%)	6 (7.7)	—	—
ICU admission, n (%)	74 (94.9)	250 (91.9)	0.53
Length of ICU stay (days)	12 (6–18)	2 (2–3)	<0.0001
28-day mortality, n (%)	10 (12.8)	0 (0.0)	<0.0001

IVR, interventional radiology; ICU, intensive care unit; FAST, focused assessment with sonography in trauma.

^α^Cases were defined as patients who required immediate intervention for hemorrhage. Immediate intervention was defined as immediate surgical/radiological intervention for hemostasis and/or blood transfusion for traumatic hemorrhage within 24 h after emergency room arrival.

^β^Data for lactate were obtained at the scene. ^†^Delta value for lactate was calculated with the following formula: (lactate in the emergency room - lactate at the scene)/time from scene to hospital. ^‡^Examination was performed at the scene. ^§^Total volume within 24 h of emergency room arrival. ^¶^Transfusion with ≥10 units of packed red blood cells.

Data are presented as median and interquartile range for continuous variables. *P* values were calculated using Pearson’s chi-square test, Fisher’s exact test, or the Mann-Whitney *U* test.

### Prediction of need for immediate intervention for hemorrhage

**Figure 1:**
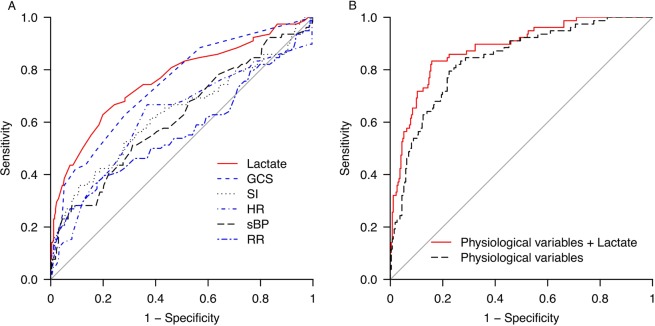
Receiver operating characteristic curve for prediction of immediate need for intervention for hemorrhage. The value of a model using potential predictors of the need for immediate intervention for hemorrhage was determined. Each physiologic parameter (sBP, HR, RR, GCS, and SI) and Lac *scene* was used for prediction in univariate analysis (**A**). Lac *scene* had the greatest predictive value (AUC = 0.764), followed by GCS (AUC = 0.744). The predictive ability of a model using a combination of physiological parameters and a penetrating mechanism of injury, with or without Lac *scene*, was estimated using multivariate analysis (**B**). Compared with use of parameters without Lac *scene* (AUC = 0.837), a combination of physiological parameters, a penetrating mechanism of injury, and Lac *scene* enabled more accurate prediction (AUC = 0.882).

In the univariate analysis of immediate need for intervention for hemorrhage, Lac *scene* had the highest area under the curve (AUC) value (0.764 [95% confidence interval (CI) 0.698–0.829]) (Table 2A) (Fig. 1A). In the primary analysis, physiological variables plus Lac *scene* significantly increased the predictive value for the need for immediate intervention for hemorrhage (AUC 0.882 [95% CI 0.839–0.925]) compared to that using physiological variables only (AUC 0.837 [95% CI 0.787–0.887]) (physiological variables vs. physiological plus lactate, *P* = 0.0073) (Table 2B) (Fig. 1B).

**Table 2:** Receiver operating characteristic curve analysis for prediction of need for blood transfusion or hemostatic intervention using each factor at the scene in cohort 1.

A. Univariate					
Variable	AUC (95% CI)	*P* value	Cut-off value	Sensitivity, specificity	OR (95% CI)
Lactate level at the scene	0.764 (0.698–0.829)	<0.0001	2.8	0.628, 0.801	6.82 (6.54–7.10)
Systolic blood pressure	0.568 (0.486–0.649)	0.22	101	0.269, 0.926	4.64 (4.30–4.99)
Heart rate	0.619 (0.544–0.694)	0.019	112	0.282, 0.919	4.46 (4.13–4.80)
Respiratory rate	0.621 (0.544–0.699)	0.020	24	0.667, 0.632	3.44 (3.17–3.71)
Glasgow Coma Scale	0.744 (0.682–0.806)	<0.0001	13	0.628, 0.717	4.28 (4.01–4.55)
Shock index	0.634 (0.556–0.712)	0.010	0.90	0.359, 0.893	4.69 (4.39–4.80)
**B. Multivariate**					
**Variable**	**AUC (95% CI)**	***P*** **value**		**Sensitivity, specificity**	**OR (95% CI)**
Physiological variables^α^	0.837 (0.787–0.887)	<0.0001		0.795, 0.776	13.03 (12.71–13.34)
Physiological variables^α^ plus lactate level at the scene	0.882 (0.839–0.925)	<0.0001		0.833, 0.842	25.60 (25.26–25.94)

AUC, area under the curve; CI, confidence interval; OR, odds ratio.

^α^Physiological variables include systolic blood pressure, heart rate, respiratory rate, Glasgow Coma Scale score, shock index score at the scene, and mechanism of penetrating injury.

In validation analysis for predictive ability in cohort 2 using the cut-off values of the derivation analysis in cohort 1 (Baseline patient characteristics and outcomes, (Table E1, see Additional file 1), the sensitivity and specificity using the combination of physiological variables plus Lac *scene* were 0.846 and 0.800, replicating the findings in cohort 1 (sensitivity = 0.833, specificity = 0.842).

### Prediction of need for massive transfusion

Univariate analysis in combined cohorts 1 + 2 (Baseline patient characteristics and outcomes, (Table E2, see Additional file 1) showed that the GCS score had the highest AUC value for prediction of the need for massive transfusion, followed by the Lac *scene* value (Table 3A**)**. In multivariate analysis of the need for massive transfusion, the predictive AUC value (0.895 [95% CI 0.846–0.944]) using physiological variables was already high (Table 3B**)**. There was no significant improvement in predictive value for massive transfusion using physiological variables plus Lac *scene* compared to that using physiological variables alone (physiological variables vs. physiological variables plus lactate, *P* = 0.32).

**Table 3:** Receiver operating characteristic curve analysis for prediction of need for massive transfusion using each factor at the scene in cohort 1 + 2.

A. Univariate					
Variable	AUC (95% CI)	*P* value	Cut-off value	Sensitivity, specificity	OR (95% CI)
Lactate	0.764 (0.661–0.867)	<0.0001	3.1	0.647, 0.835	9.08 (8.70–9.46)
Systolic blood pressure	0.713 (0.607–0.819)	0.00073	93	0.441, 0.955	16.48 (16.06–4.01)
Heart rate	0.651 (0.539–0.764)	0.023	100	0.529, 0.766	3.65 (3.29–4.01)
Respiratory rate	0.530 (0.406–0.654)	0.73	24	0.618, 0.576	2.16 (1.80–2.52)
Glasgow Coma Scale	0.796 (0.718–0.874)	<0.0001	8	0.559, 0.910	12.60 (12.22–12.98)
Shock index	0.763 (0.663–0.862)	<0.0001	0.95	0.529, 0.910	11.23 (10.85–11.61)
**B. Multivariate**					
**Variable**	**AUC (95% CI)**	***P*** **value**		**Sensitivity, specificity**	**OR (95% CI)**
Physiological variables^α^	0.895 (0.846–0.944)	<0.0001		0.853, 0.820	24.38 (23.89–24.86)
Physiological variables^α^ plus lactate level at the scene	0.903 (0.851–0.956)	<0.0001		0.941, 0.748	38.48 (37.82–39.15)

AUC, area under the curve; CI, confidence interval; OR, odds ratio.

^α^Physiological variables include systolic blood pressure, heart rate, respiratory rate, Glasgow Coma Scale score, shock index score at the scene, and positive mechanism of penetrating injury.

### Change in blood lactate levels from scene to ER (Lac delta per min)

**Figure 2:**
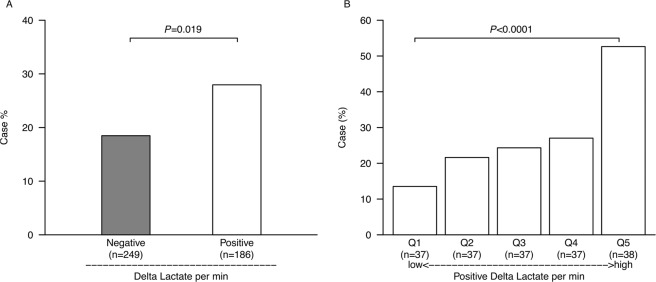
Case probability of patients with early therapeutic intervention in Lac *delta per min* groups. There was a significant difference in case probability between the negative Lac *delta per min* group (n = 249) and the positive Lac *delta per min* group (n = 186) (**A** *P = 0.019 with the chi-square test). In quintile subgroups of the positive Lac *delta per min* group, the case probability was significantly increased with increasing Lac *delta per min* (**B** **P < 0.0001 with the Cochran-Armitage test).

Patients in the positive Lac *delta per min* group, with increased blood lactate levels from scene to ER, had significantly increased probability of need for immediate intervention for hemorrhage compared to the negative Lac *delta per min* group (*P* = 0.019) (Fig. 2A). In the positive Lac *delta per min* group, the case probability was significantly increased in higher quintiles (*P* < 0.0001) (Fig. 2B).

## Discussion

This study investigated the predictive value of blood lactate levels at the scene for the need for immediate intervention for hemorrhage. We found that adding the blood lactate level at the scene to a combination of physiological variables significantly improved the predictive value. In contrast, the combination of physiological variables without blood lactate levels showed high predictive value for the need for massive transfusion; there was no significant improvement in predictive value using physiological variables plus Lac *scene* for the need for massive transfusion compared to that using physiological variables alone. However, the increased blood lactate level per minute from scene to ER was associated with increased probability of the need for immediate intervention for hemorrhage.

The potential usefulness of prehospital lactate was suggested as a tool for urgent ambulance dispatch for patients with circulatory, respiratory, or neurological disorders^12^, as well as a predictive tool for resuscitative care^13^ and designation of trauma activation level^14^. Since the prehospital lactate cost per measurement is not large ($2.00 per measurement) in the study, the study results have high translatability into clinical practices. In addition, lactate data on ER arrival may be useful for initial in-hospital triage to determine which patients need early trauma team activation and surgical intervention^15^. Massive transfusion only occurs in 1% to 5% of civilian trauma patients^6^; the rate of immediate intervention for hemorrhage is higher than that for massive transfusion. Furthermore, more deaths occur in patients who receive <10 units of packed RBCs^7^. Thus, we focused on immediate intervention for hemorrhage in the current study. In addition, since predicting which patients will have less hemorrhage may be difficult, developing predictive algorithms with high precision based on data at the earliest phase may improve the quality of subsequent trauma care.

In a study of blood transfusion with ≥6 units of packed RBCs within 24 h, prehospital blood lactate level had a better predictive value than sBP for transfusion requirement (AUC, lactate vs. sBP, 0.72 vs 0.61)^16^. We also found that prehospital blood lactate level had better predictive value for the need for immediate intervention for hemorrhage compared to that using sBP (AUC, lactate vs. sBP, 0.764 vs. 0.568). In normotensive trauma patients, there was also a significant association between prehospital lactate and transfusion requirements; however, the predictive value of a single prehospital lactate level was inadequate (AUC = 0.68). Thus, a single lactate level may be insufficient to predict the need for transfusion or immediate intervention for hemorrhage. In the primary analysis of the study, we found that the blood lactate level at the scene in combination with physiological variables significantly improved the predictive value (Table 2B). The predictive ability was replicated in validation analysis. The predictive value of physiological variables plus lactate level at the scene was very high (AUC 0.882, 95% CI 0.839–0.925), and highlights the importance of lactate measurement combined with physiological variables. It was reported that the assessment of blood consumption (ABC) score, which only includes sBP, HR, a positive focused assessment with sonography in trauma (FAST) and a penetrating mechanism of injury without blood test, have high predictive value for the need for massive transfusion (AUC, 0.83–90)^4,5,9^. This study similarly found that use of physiological variables alone had a very high predictive value (AUC 0.895) (Table 3B). Thus, physiologic parameters may have sufficiently high predictive value for the need for massive transfusion. In addition, we found no significant improvement in predictive value with use of physiological variables plus Lac *scene* for the need for massive transfusion compared to that using physiological variables alone. Another study similarly showed that either continuous pulse oximetry or prothrombin time could be more useful in prediction of the need for massive transfusion than lactate levels^17^. Furthermore, a predictive model using 15-min continuous vital sign data had high predictive value for the need for massive transfusion (AUC 0.91)^18^. Thus, continuous monitoring of physiologic parameters may have better predictive value for the need for massive transfusion.

The utility of blood lactate measurement in the ER for patients with trauma and sepsis has been widely recognized^19,20^. Delta lactate, also called lactate clearance, is useful in evaluating the trend in circulatory failure in trauma^21^ and sepsis^22^. In this study, the positive Lac *delta per min* group had a significantly increased case probability compared to that in the negative Lac *delta per min* group. In addition, in the positive Lac *delta per min* group, the case probability was significantly increased in higher quintiles. Thus, in addition to the use of prehospital lactate at the scene for prediction of the need for immediate intervention, assessing the amount of change on ER arrival may detect patients who had bleeding during transfer and need immediate intervention for hemorrhage.

This study had several limitations. While demonstrating the significant value of blood lactate levels at the scene, this observational study was retrospectively conducted at a single level I trauma center. Although there was a significant association between Lac *delta per min* and altered case probability, the transfer time from scene to hospital was approximately 20 min, due to the urban location. Further studies in different settings may strengthen the findings for the value of Lac *delta per min*.

## Conclusions

Adding blood lactate levels at the scene to a predictive algorithm using physiological variables significantly improves the predictive value of the need for immediate intervention for hemorrhage. The increase in blood lactate levels from scene to ER was associated with a high probability of the need for immediate intervention. Measuring blood lactate level at the scene appears to be a useful option in trauma care.

## Methods

The institutional review board at Senshu Trauma and Critical Care Center approved this study. The methods were carried out in accordance with the Declaration of Helsinki and the relevant guidelines. The review board waived the need for written informed consent from subjects or their legal surrogates.

### Study setting and patients

We prospectively measured blood lactate levels and assessed retrospectively. However, the current observational study was retrospectively conducted and may have potential biases.

The Senshu Trauma and Critical Care Center is a level I regional trauma center located in an urban area, Osaka, Japan, and covers about 1 million residents within a 40-km radius. The trauma center has a fully-equipped ambulance to provide advanced prehospital care by trained trauma physicians 24/7. When regional 911 centers receive trauma calls that meet predetermined criteria for severe injury, the emergency ambulance is dispatched, staffed with 2 trained trauma physicians, 1 nurse, 1 EMS technician, and a driver^23^.

Consecutive trauma patients (n = 1,695) who were transferred to the center between April 2014 and September 2017 were screened. Of these, 616 patients who were transferred via the trauma physician-staffed ambulance were included. Of these, 94 patients with seizures, hypothermia (below 35.0 °C), acute alcohol/drug intoxication, or cardiac arrest were excluded, as these conditions potentially increase blood levels of lactate. Of the remaining 522 patients, 72 without data for blood lactate levels were excluded. Thus, 435 patients were eligible for the analysis (Fig. E1, see Additional file 1).

To demonstrate robustness of the present study findings, we categorized subjects into a derivation cohort 1 or validation cohort 2. According to a sample size calculation based on area under the curve (AUC) values in previous trauma studies on transfusion (AUC = 0.80, power = 0.95, significance = 0.05, pROC package for R)^4,17,24^, the required sample size in a validation cohort was n = 20 per group. To obtain the required sample size in a validation cohort, study subjects were randomly divided into a derivation cohort 1 (total n = 350; case = 78, control = 272) and a validation cohort 2 (total n = 85; case = 20, control = 65) using the rand function of MATLAB software package (version 8.3.0.532 (R2014a), MathWorks, Inc., Natick, MA, USA).

### Data collection and definition

Physiologic data collected by a trained trauma team (nurse and physician) on scene included: systolic blood pressure (sBP), heart rate (HR), respiratory rate (RR), Glasgow Coma Scale (GCS) score, and shock index (SI) calculated with sBP divided by HR.

Blood levels of lactate at the scene (Lac *scene*) were immediately measured after arrival using a portable device (Lactate Pro, Arkray, Kyoto, Japan). The test strip fills with 5 μL of whole blood through capillary action and takes 60 s to provide the lactate level. The cost of test strip per measurement is approximately $2.00. A drop of venous blood from a peripheral catheter immediately after insertion and before connection of infusion fluid was used for lactate measurement. Blood levels of lactate in the ER (Lac *emergency room*) were measured immediately after arrival at the trauma center using a blood gas analyzer (ABL800, Radiometer, Copenhagen, Denmark). A strong correlation between blood levels of lactate using the two devices was previously reported^25^.

The change in amount of blood lactate per minute between Lac *emergency room* and Lac *scene* was defined as Lac *delta per min*: (Lac *emergency room* - Lac *scene*) / time in minutes from medical team arrival at the scene to emergency room arrival. The “negative Lac *delta per min* group” was defined by Lac *delta per min* < 0 and the “positive Lac *delta per min* group” was defined by Lac *delta per min* ≥ 0.

The primary outcome variable was immediate intervention for hemorrhage, defined as immediate surgical/radiological intervention for hemostasis and/or blood transfusion. Immediate hemostatic intervention was defined as intervention immediately after assessment in the ER and included either surgical or radiological intervention for hemostasis. Immediate blood transfusion was defined as blood transfusion for traumatic hemorrhage within 24 h after ER arrival. We categorized patients with immediate interventions for hemorrhage into the case group and those without interventions into the control group.

The secondary outcome variable was massive transfusion. Massive transfusion was defined as ≥10 units of packed RBCs in the first 24 h^8^.

### Statistical analysis

Each of the physiological variables (sBP, HR, RR, GCS, and SI) and Lac *scene* was analyzed as a determinant with univariate logistic regression in cohort 1. Multivariate logistic regression analysis was simultaneously performed. A penetrating mechanism of injury was reported in predictive algorithms for transfusion^4^, and was added in the multivariate analysis as a covariate. The predictive ability of each model was evaluated with a receiver operating characteristic (ROC) curve and corresponding AUC, which were derived through leave-one-out cross-validation. We determined the optimal cut-off points with the Youden index to estimate the sensitivity, specificity, and odds ratio for the factors in each regression model.

For primary analysis of the study, we compared the ROC-AUC results for physiological variables only with physiological variables plus Lac *scene* using the DeLong test. To validate the results in cohort 1, we analyzed the sensitivity, specificity, and odds ratio (OR) in combination with the physiological variables and lactate in cohort 2 using the cut-off points of cohort 1.

A secondary analysis in the study assessed need for massive transfusion. The predictive ability for massive transfusion was analyzed using the same methods for immediate intervention for hemorrhage. Due to a small sample size, we combined cohorts 1 and 2 to analyze use of massive transfusion.

Another secondary analysis examined Lac *delta per min*. We first compared the case probability between negative and positive Lac *delta per min* groups using the chi-square test. We further compared the case probability among quintile subgroups in the positive Lac *delta per min* group using the Cochran-Armitage test.

We tested for differences in baseline characteristics using the chi-square test or Fisher’s exact test for categorical data and the Mann-Whitney *U* test for continuous data. Data were expressed as median (interquartile range [IQR]) for continuous values and absolute number and percentage for categorical values. A two-tailed *P* value < 0.05 was considered significant. Analyses were performed using R (version 3.3.2, www.R-project.org) and pROC package for R was used for sample size calculation.

### Ethics approval and consent to participate

The institutional review board at Senshu Trauma and Critical Care Center approved this study. The methods were carried out in accordance with the Declaration of Helsinki and the relevant guidelines. The review board waived the need for written informed consent from subjects or their legal surrogates.

## Supplementary information

Additional file 1

## Data Availability

The datasets used and analyzed during our study are available from the corresponding author upon reasonable request.
