# Supplementary material for: Prehospital lactate improves prediction of the need for immediate interventions for hemorrhage after trauma
Source: Sci Rep. 2019 Sep 24;9:13755. doi: 10.1038/s41598-019-50253-6 (PMC6760524; doi:10.1038/s41598-019-50253-6)

Prehospital lactate improves prediction of the need for immediate interventions for hemorrhage after trauma

Hiroshi Fukuma, Taka-aki Nakada, Tadanaga Shimada, Takashi Shimazui, Tuerxun Aizimu, Shota Naka, Hiroaki Watanabe, Yasuaki Mizushima, Tetsuya Matsuoka

Supplemental Digital Content

Table E1. Baseline patient characteristics and clinical outcomes in cohort 2

Characteristics or outcomes	Case[α] (n=20)	Control (n=65)	*P*-values
Age, years	56 (42-63)	42 (22-59)	0.034
Male, n (%)	13 (65.0)	59 (90.8)	0.010
Mechanism of injury			
Penetrating, n (%)	2 (10.0)	0 (0.0)	0.053
Blunt, n (%)	18 (90.0)	65 (100.0)	0.69
Road injury, n (%)	14 (70.0)	53 (81.5)	
Fall, n (%)	4 (20.0)	10 (15.4)	
Compression machinery, n (%)	0 (0.0)	2 (3.1)	
Other, n (%)	0 (0.0)	0 (0.0)	
Physiological data[β]			
Systolic blood pressure, mm Hg	121 (73-134)	138 (119-155)	0.0018
Heart rate, beats/min	91 (76-112)	82 (72-90)	0.10
Respiratory rate, breaths/min	24 (14-30)	20 (18-25)	0.50
Glasgow Coma Scale	8 (5-13)	14 (14-15)	<0.0001
Shock index	0.79 (0.64-1.20)	0.59 (0.53-0.76)	0.0018
Lactate			
Scene, mg/dL	3.2 (2.2-5.2)	1.7 (1.2-2.6)	<0.0001
Emergency room, mg/dL	3.8 (2.2-7.1)	1.9 (1.1-2.4)	<0.0001
Delta[†], delta/min	-0.015 (-0.066-0.081)	0.00 (-0.013-0.014)	0.75
Scene to hospital, min	21 (14-26)	18 (15-24)	0.83
Injury severity score	32 (22-42)	5 (1-14)	<0.0001
Positive FAST exam[‡], n (%)	4 (20.0)	0 (0.0)	0.0024
Blood transfusion, n (%)	20 (100.0)	-	-
Red blood cell[§], mL	280 (0-840)	-	-
Fresh frozen plasma[¶], mL	1080 (720-1680)	-	-
Massive transfusion, n (%)	6	-	
Hemostatic intervention			
Surgery, n (%)	4 (20.0)	-	-
IVR, n (%)	5 (25.0)	-	-
Both, n (%)	1 (5.0)	-	-
ICU admission, n (%)	20 (100.0)	62 (95.4)	1.00
Length of ICU stay (days)	10 (8-16)	2 (2-3)	<0.0001
28-day mortality, n (%)	3 (15.0)	1 (1.5)	0.038

IVR, interventional radiology; ICU, intensive care unit; FAST, focused assessment with sonography in trauma.
[α] Cases were defined as patients who required blood transfusion or hemostatic intervention. [β] Data for lactate were obtained at the scene. [†] Delta value for lactate was calculated using the following formula: (lactate in the emergency room - lactate at the scene)/time from scene to hospital. [‡] Examination was performed at the scene.' [§] Total volume within 24 h of emergency room arrival. [¶] Transfusion with ≥ 10 units of packed red blood cells.

Data are presented as median and interquartile range for continuous variables. *P* values were calculated using Pearson’s chi-square test, Fisher's exact test, or the Mann-Whitney *U* test.

Table E2. Baseline patient characteristics and clinical outcomes in cohort 1 + 2

Characteristics or outcomes	Massive transfusion[α] (n=34)	Control (n=401)	*P*-values
Age, years	64 (45-77)	42 (23-59)	<0.0001
Male, n (%)	20 (58.8)	282 (70.3)	0.162
Mechanism of injury			
Penetrating, n (%)	0 (0)	12 (3.0)	0.301
Blunt, n (%)	34 (100)	389 (97.0)	0.298
Road injury, n (%)	26 (76.5)	322 (80.3)	
Fall, n (%)	7 (20.6)	43 (10.7)	
Compression machinery, n (%)	1 (2.9)	11 (2.7)	
Other, n (%)	0 (0)	13 (3.2)	
Physiological data[β]			
Systolic blood pressure, mm Hg	112 (74-141)	132 (118-149)	<0.0001
Heart rate, beats/min	100 (81-126)	85 (74-98)	0.01
Respiratory rate, breaths/min	24 (12-30)	21 (16-26)	0.97
Glasgow Coma Scale	8 (4-13)	14 (13-15)	<0.0001
Shock index	0.98 (0.71-1.33)	0.64 (0.54-0.77)	0.0001
Lactate			
Scene, mg/dL	4.0 (2.6-5.2)	2.1 (1.6-2.8)	<0.0001
Emergency room, mg/dL	4.0 (2.9-8.7)	1.9 (1.3-2.8)	<0.0001
Delta[†], delta/min	0.7 (-0.1-2.7)	-0.1 (-0.5-0.4)	0.004
Scene to hospital, min	18 (14-24)	18 (14-26)	0.164
Injury severity score	34 (26-45)	10 (2-21)	<0.0001
Hemostatic intervention			
Surgery, n (%)	5 (14.7)	9 (2.2)	<0.0001
IVR, n (%)	13 (38.2)	20 (5.0)	<0.0001
Both, n (%)	5 (14.7)	1 (5.0)	<0.0001
ICU admission, n (%)	33 (97.1)	373 (93.0)	0.364
Length of ICU stay (days)	17 (6-23)	2 (2-5)	<0.0001
28-day mortality, n (%)	9 (26.5)	5 (1.2)	<0.0001

IVR, interventional radiology; ICU, intensive care unit
[α] Transfusion with ≥ 10 units of packed red blood cells. [β] Data for lactate were obtained at the scene. [†] Delta value for lactate was calculated with the following formula: (lactate in the emergency room - lactate at the scene)/time from scene to hospital.
Data are presented as median and interquartile range for continuous variables. *P* values were calculated using Pearson's chi-square test, Fisher's exact test, or the Mann-Whitney *U* test.

Figure E1. Patient flow diagram

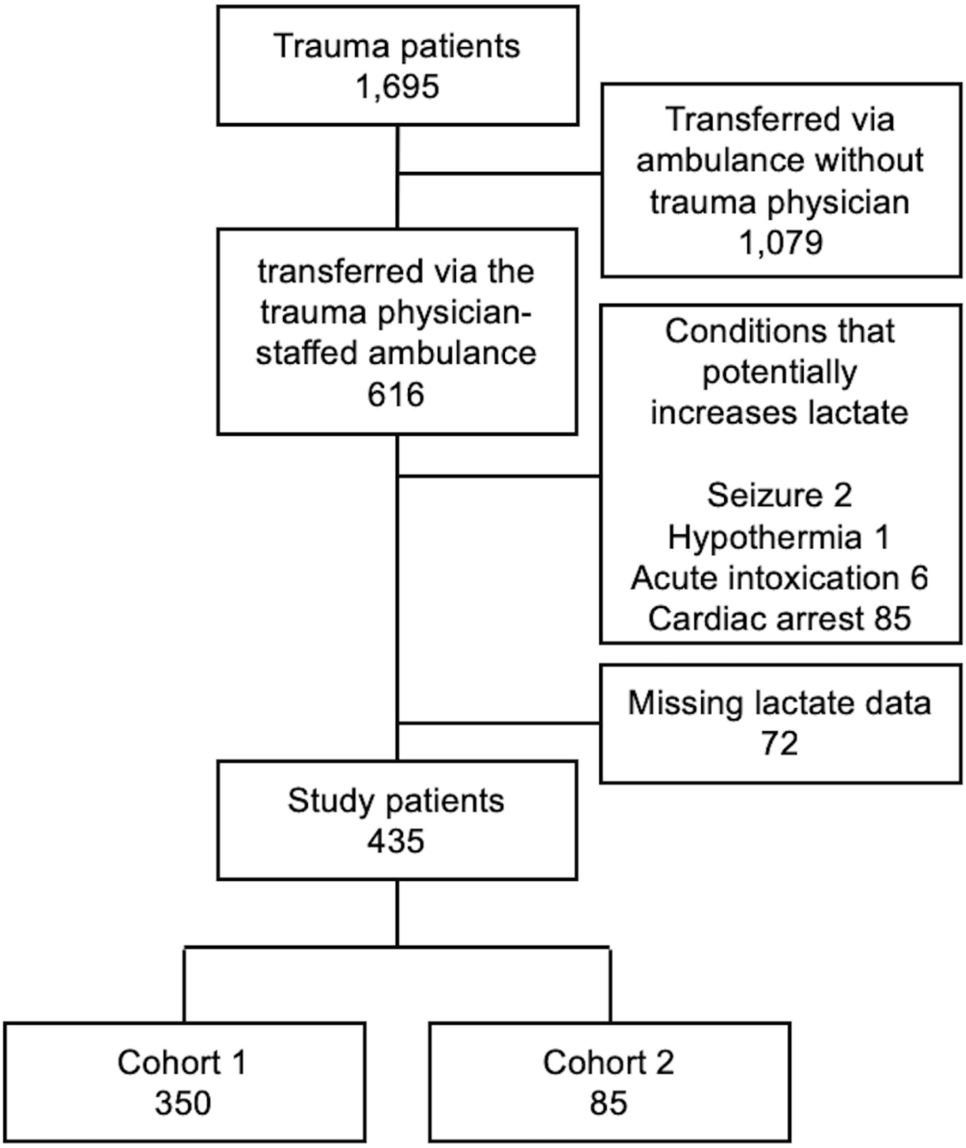

Supplement: Supplementary file 1 — Additional file 1 [file 41598_2019_50253_MOESM1_ESM.pdf]
